# Hirschsprung’s Disease: A Rare Adult Diagnosis

**DOI:** 10.5811/cpcem.2020.6.46492

**Published:** 2020-07-30

**Authors:** Kaitlyn Schmutz, Gaea McGaig, B. Jason Theiling

**Affiliations:** * Duke University Hospital, Department of Emergency Medicine, Durham, North Carolina; † Rex Hospital, Department of Emergency Medicine, Raleigh, North Carolina; ‡ Duke University School of Medicine, Department of Surgery, Division of Emergency Medicine, Durham, North Carolina

**Keywords:** Hirschsprung’s disease, chronic constipation

## Abstract

**Case Presentation:**

Approximately 94% of patients with Hirschsprung’s disease (HD) are diagnosed before the age of five. In our case, a young adult with years of constipation presented to the emergency department with significant abdominal distention. He was ultimately diagnosed with HD, which was identified using computed tomography (CT).

**Discussion:**

In HD, we find defects in gastric motility due to improper gut colonization. Without childhood recognition, HD often leads to chronic constipation and failure to thrive in adulthood. CT is a key step in identifying this rare adult diagnosis that should be considered in all patients with a history of chronic constipation.

## CASE PRESENTATION

**Image 1:**
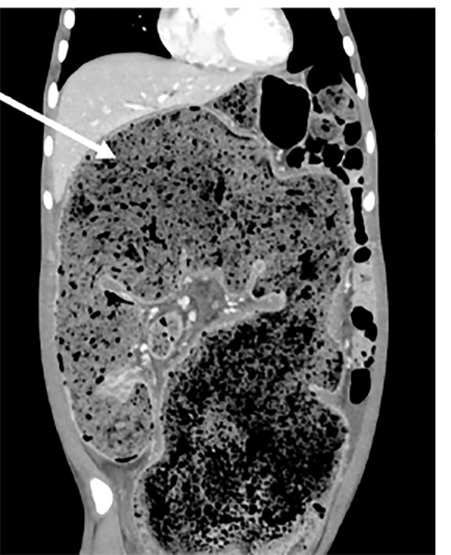
Sagital computed tomography with intravenous contrast demonstrating marked colonic distention with large stool burden.

**Image 2:**
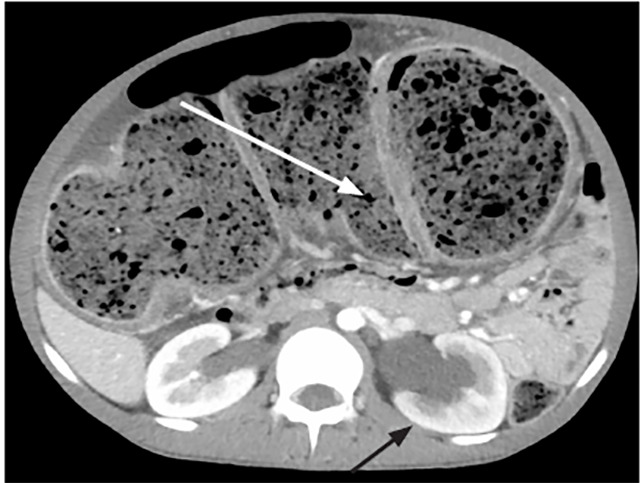
Coronal computed tomography with intravenous contrast demonstrating marked colonic distention (white arrow) and hydronephrosis (black arrow).

An 18-year-old male with history of chronic constipation (CC) presented to the emergency department complaining of left lower extremity (LLE) swelling and abdominal distention. Despite a daily polyethylene glycol regimen, he had previously required both manual and procedural disimpactions. He was tolerating both solids and liquids without vomiting. He denied infectious symptoms and was afebrile. On examination, his abdomen was distended without tenderness. The LLE had circumferential pitting edema without erythema or tenderness. He underwent computed tomography (CT) of the abdomen and pelvis with intravenous contrast that demonstrated a high degree of colonic distention (Image 1) with mass effect causing hydronephrosis, intrahepatic biliary ductal dilatation, and mesenteric venous engorgement (Image 2).

There was also CT evidence of iliac vein compression (left greater than right) that caused his LLE edema. He ultimately underwent colonic decompression followed by colonoscopy and rectal biopsy, which confirmed his diagnosis of Hirschsprung‘s disease (HD).

## DISCUSSION

HD occurs in 1:5000 births, but in adults it is rarely considered and often undiagnosed. The pathophysiology of HD is an absence of intramural ganglion cells of the submucosal (Meissner’s) and myenteric (Auerbach’s) neural plexuses, which are situated between smooth muscle layers in the affected bowel segment.^1^,^2^ While it is likely that the colonic region proximal to the distal obstructed segment assumes a compensatory role in function for undiagnosed adults, these patients will often still suffer from CC.^3^ CC has prevalence estimates from 1%–8% in North America with significant impact on quality of life.^4^ A CT suggestive of HD could lead to complete eradication or significant improvement in CC by confirmational biopsy and definitive surgical management.^5^ For these reasons, Hirschsprung’s disease should be considered in all adults with refractory constipation.

**CPC-EM Capsule**

What do we already know about this clinical entity?

Hirschsprung’s disease (HD) is characterized by gastric dysmotility and is associated with neonates.

What is the major impact of the image(s)?

This computed tomography of a young adult patient demonstrates colonic distention with significant mass effect, which was highly suspicious for undiagnosed HD.

How might this improve emergency medicine practice?

While it is a congenital condition, HD can present later in life as chronic constipation and should prompt an expansion of the differential diagnosis.
